# (In)validating experimentally derived knowledge about influenza A defective interfering particles

**DOI:** 10.1098/rsif.2016.0412

**Published:** 2016-11

**Authors:** Laura E. Liao, Shingo Iwami, Catherine A. A. Beauchemin

**Affiliations:** 1 Department of Physics, Ryerson University, Toronto, Canada; 2 Department of Biology, Kyushu University, Fukuoka, Japan; 3 CREST and PRESTO, Japan Science and Technology Agency (JST), Saitama, Japan; 4 Interdisciplinary Theoretical Science (iTHES) Research Group at RIKEN, Wako, Japan

**Keywords:** defective interfering particles, influenza A virus, interference assay, reduction of infectious virus yield, mathematical model, co-infection

## Abstract

A defective interfering particle (DIP) in the context of influenza A virus is a virion with a significantly shortened RNA segment substituting one of eight full-length parent RNA segments, such that it is preferentially amplified. Hence, a cell co-infected with DIPs will produce mainly DIPs, suppressing infectious virus yields and affecting infection kinetics. Unfortunately, the quantification of DIPs contained in a sample is difficult because they are indistinguishable from standard virus (STV). Using a mathematical model, we investigated the standard experimental method for counting DIPs based on the reduction in STV yield (Bellett & Cooper, 1959, *Journal of General Microbiology* **21**, 498–509 (doi:10.1099/00221287-21-3-498)). We found the method is valid for counting DIPs provided that: (i) an STV-infected cell's co-infection window is approximately half its eclipse phase (it blocks infection by other virions before it begins producing progeny virions), (ii) a cell co-infected by STV and DIP produces less than 1 STV per 1000 DIPs and (iii) a high MOI of STV stock (more than 4 PFU per cell) is added to perform the assay. Prior work makes no mention of these criteria such that the method has been applied incorrectly in several publications discussed herein. We determined influenza A virus meets these criteria, making the method suitable for counting influenza A DIPs.

## 1. Introduction

**Figure 1.**
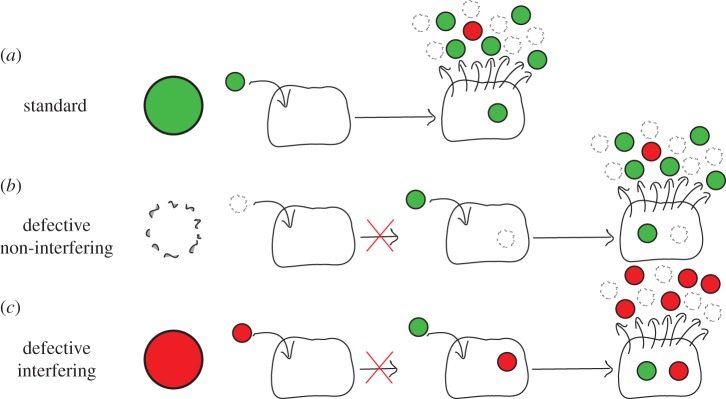
Process of standard virus yield suppression by DIPs. (*a*) A cell infected by standard (non-defective) influenza A virus (STV) yields virus progeny comprising STV, DIPs and defective non-interfering particles. (*b*) Defective non-interfering particles are either unable to enter susceptible cells, or can enter cells but are replication-incompetent in a manner that does not interfere with further infection by STV. (*c*) By contrast, DIPs can infect cells, but only upon additional infection with STV can the co-infected cell produce progeny, though DIPs will be produced at the expense of STV in these cells, resulting in suppression of STV yield.

A cell that is infected with standard virus, will produce progeny that is a mixture of infectious, functional virus—hereafter referred to as standard virus (STV)—and particles that are defective (figure 1*a*) because viral replication is an error-prone process, especially for RNA viruses. Defective, virus-like particles that either cannot initiate infection or can but are replication-incompetent are referred to as defective particles. If these defective particles do not interfere with STV replication, they are called defective non-interfering particles [1] (figure 1*b*). By contrast, defective particles that can initiate infection, but are replication-incompetent in a manner that causes them to interfere with STV replication, are known as defective interfering particles (DIPs) (figure 1*c*). The properties of DIPs have been enumerated in [2]. Namely, they resemble STV because they are composed of the same structural proteins, they contain an incomplete copy of the viral genome, and they require STV to replicate.

Owing to differences in the mechanisms of infection and replication of different viruses, what constitutes a DIP, i.e. the factors that can cause a defective particle to interfere with the replication of its STV counterpart, will vary across viruses. Herein, our results and analyses focus on DIPs in the context of the influenza A virus. Influenza A virions have a segmented genome comprising eight negative-sense viral RNA (vRNA) segments packed within a capsid, enveloped by a host membrane containing embedded viral proteins. An influenza A DIP is identical to its STV counterpart, however, at least one of its eight vRNA segments have been replaced by a defective interfering (DI) vRNA segment (for a review, refer to [3]). When interference has been observed, influenza A DI vRNAs were shown to contain a large internal deletion making them shorter than their corresponding full-length parent segment [4]. Influenza A DI vRNAs originating from vRNA segments that encode for viral polymerase (PB2, PB1 and/or PA) have been observed most frequently [4,5], though influenza A DI vRNAs originating from other segments have also been observed [6].

On short timescales, the dynamics of DIPs is governed by two processes shown in figure 1: de novo generation (figure 1*a*) and their amplification (figure 1*c*). A cell infected only by an influenza A DIP with a truncated polymerase vRNA segment cannot produce de novo functional viral polymerase, such that the virus replication cycle cannot be completed and will not yield any virus progeny. Upon co-infection with influenza A STV, the full-length polymerase vRNA segment contributed by the STV will be translated into functional viral polymerase, which will proceed to replicate and transcribe all segments contributed by the DIP, including its shortened DI polymerase segment, thereby successfully completing the virus replication cycle to produce progeny. It is hypothesized that more copies of the shorter influenza A DI segment are produced per unit time than the full-length segment, and that the greater number of DI segments sequesters intracellular resources, inhibiting STV replication [7]. As reviewed in [8], there are still many open questions about the mechanism of influenza A DIP interference, and the length advantage of influenza A DI vRNA is not the sole explanation for interference. For example, it has been shown that influenza A DI vRNAs are packaged more efficiently than standard vRNA [9,10]. Ultimately, a cell co-infected by influenza A DIPs and STV will produce mainly progeny DIPs at the expense of STV, resulting in significant suppression of influenza A STV yield.

**Figure 2.**
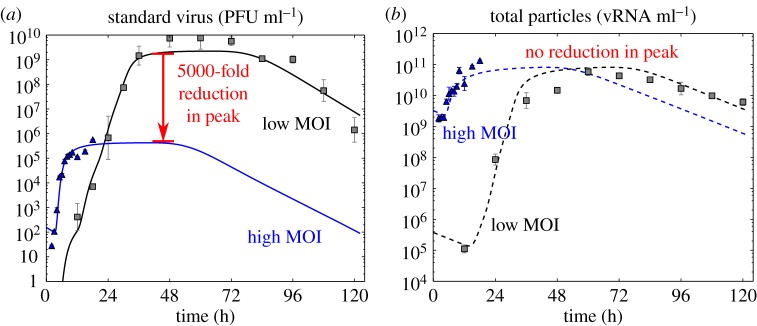
The presence of DIPs is only evident at high STV MOI. The concentration of STV (*a*) and total (STV + DIP + defective non-interfering) particles (*b*) were measured at various time points over the course of two *in vitro* infection experiments with the 2009 pandemic influenza A/Québec/144147/09 (H1N1) virus strain. The curves are the result of a previously reported mathematical analysis of these infections [11]. Both experiments were performed at the same time, in the same manner, differing only in the concentration of STV in the initial inoculum, with one conducted at a low MOI of 10^−5^ PFU per cell (grey), and the other at a high MOI of 4 PFU per cell (blue). While the peak total particles for both infections was 10^11^ vRNA ml^−1^ (*b*), a 5000-fold drop in the STV peak is observed (*a*) for infection with a high MOI. It is typically assumed that this reduction in STV yield is evidence of the presence of DIPs in the STV stock.

As the suppression of influenza A STV yield by DIPs requires the co-infection of a cell by both an STV and a DIP, the effect of DIPs is thought to be significant only in assays where the likelihood of a co-infection event is high. An experiment conducted at low multiplicity of infection (MOI), e.g. with an inoculum containing one virion per 100 000 cells, is unlikely to result in any one cell receiving two or more virions. Inoculation at a high MOI, e.g. 4 plaque forming units (PFU) per cell, however, will result in a large number of co-infected cells. This is why the presence of DIPs in an inoculum of high MOI causes a significant reduction in STV yield compared with one of low MOI. This can be seen in figure 2 where an influenza A virus infection conducted with a high MOI resulted in a 5000-fold reduction in peak STV concentration but the same total (STV + defective) particle peak concentration compared with that observed for the same experiment conducted at a low MOI. Others have also observed a reduction in influenza A STV yield due to DIPs, with no decrease in the total influenza A particles produced [12,13].

Even in an assay initiated with a highly diluted inoculum infecting only a few cells, a high rate of virion production by these cells might, at least transiently, lead to a high virus concentration in their immediate vicinity. This high local concentration could, theoretically, result in the co-infection of at least some cells. Therefore, the presence of DIPs in a sample, even in assays inoculated with a low MOI, has the potential to impact conclusions drawn from measurements of STV. For example, when comparing the virulence of strains A and B, observing lower STV yield for strain A would be thought to indicate a lower virulence. On the other hand, the lower STV yield could be due to the presence of a large proportion of DIPs in the sample for strain A which would otherwise exhibit STV yields similar to, or even greater than, that of strain B. To avoid such confounding effects, it is thought that passaging virus samples at low MOI, especially in combination with plaque purification [8,14], can reduce the proportion of DIPs in samples to sufficiently low levels so as not to interfere with common assays.

Influenza A DIPs differ from their STV counterpart only in a deletion in one of the eight influenza A vRNA segments, which are packed into the same capsid, enveloped by the same host membrane, with the same embedded proteins. This minor difference between influenza A DIPs and STV is insufficient to allow for their separation and quantification based on physical characteristics (e.g. appearance, weight, volume, charge), an issue common also to other viruses [15–19]. For this reason, in 1959, Bellett & Cooper [20] (hereafter, B&C) introduced an assay whose data can be used to compute, rather than directly measure, the content of DIPs in a sample by indirectly inferring their concentration based on the observed reduction in STV yield they cause. To this day, the B&C assay and variations thereof which rely on the same principles and assumptions, continue to be the primary manner by which DIPs are quantified [21–23]. Much of what is thought to be known about DIPs and the methodologies developed to mitigate their impact are based on inferences drawn from these indirect quantifications. Unfortunately, when performing different variations of the B&C assay, others have commonly observed deviations of their experimental data from the theoretical trend predicted by the B&C method [16,20,21,24–26]. This indicates that, at least under some conditions, the B&C calculation is invalid and its use could lead to incorrect conclusions about the presence of DIPs in a virus sample, or misguided inferences about their impact under certain assay conditions.

In this work, we revisit the B&C assay in the context of influenza A DIPs. We evaluate the extent of its validity using a mathematical model for influenza A virus infection that explicitly accounts for DIPs. We identify the assumptions made by B&C in computing the concentration of DIPs based on the reduction in infectious STV yield. We establish conditions under which the B&C assay must be performed for these assumptions to hold true. We also provide explanations and possible remedies for deviations of experimental observations in the B&C assay from the trend predicted theoretically by the B&C calculation.

## 2. Results

### 2.1. Theoretical basis and failure of the Bellett & Cooper assay

**Figure 3.**
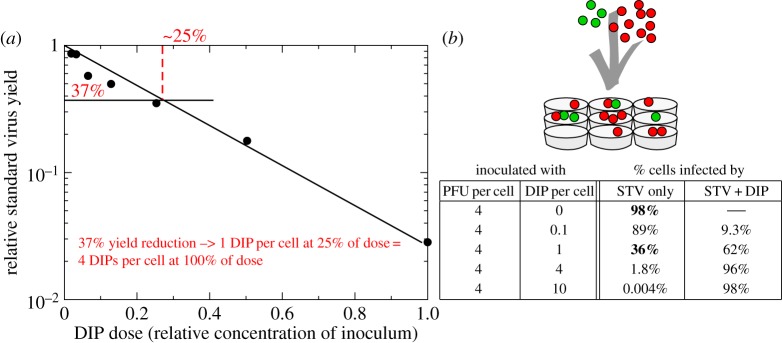
Counting of DIPs in the B&C 1959 assay. (*a*) In the B&C experiment, parallel cell cultures were incubated for 1 h with an inoculum containing a fixed, known concentration of a DIP-free standard VSV stock plus increasing dilutions of a VSV sample of unknown DIP concentration, and then rinsed. The STV yield (in PFU ml^−1^) in the cell cultures' supernatant was determined at 20 h post-rinsing, and expressed relative to the STV yield in the absence of DIPs (*y*-axis) as a function of decreasing dilutions (increasing DIP doses, *x*-axis) of the virus sample (data taken from figure 1 in [20]). (*b*) When inoculating nine cells with 4 STV (PFU, green) and 10 DIPs (red), a percentage of cells will not be infected while others will be infected by one or more STV and/or DIP. These percentages follow a Poisson process and can be computed (see electronic supplementary material, section S5) for any number of PFU and DIPs in an inoculum. Assuming that only cells infected by STV alone produce STV, a relative STV yield of 37% when the sample is diluted in a ratio of 1 : 4 (25%) is thought to indicate that the sample DIP MOI was 1 DIP per cell at this dilution, or 4 DIPs per cell when undiluted.

In 1959, B&C performed an assay, which exploits the reduction of STV yield observed in the presence of DIPs, to determine the concentration of DIPs in a sample of vesicular stomatitis virus (VSV). They infected parallel cell cultures with an inoculum consisting of a known, fixed concentration of a pure, DIP-free standard VSV stock, mixed with increasing dilutions of a sample containing an unknown concentration of standard VSV and DIPs. The results of this important experiment are shown in figure 3*a*, where a marked decrease in the STV yield can be seen for increasing concentrations of their DIP-containing VSV sample. From these data, the authors concluded that, at their chosen volume of inoculum per cell, their undiluted sample contained DIPs at an MOI of 4 DIPs per cell. Their conclusion relies on a number of simple, but seemingly reasonable steps.

As illustrated in figure 3*b*, given a particular MOI of DIP and STV per cell, the distribution of these particles within a population of cells is statistically described by the Poisson distribution. Hence, from the number of each type of infecting particle (STV, DIP) it is possible to theoretically calculate the fraction of cells infected only with DIPs or STV, co-infected by both, or uninfected (for this calculation, see electronic supplementary material, section S5). For example, inoculating cells with 4 PFU per cell and no DIPs will result in 98% of cells infected by STV alone, while inoculating with 4 PFU per cell + 1 DIP per cell will result in 36% STV-only infected cells, or 37% 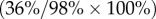 of that in the absence of DIPs. B&C further assume that the STV yield is proportional to the fraction of STV-only infected cells, i.e. they assume that STV-only infected cells are the sole significant producers of STV. Thus, according to B&C, the reduction in STV yield corresponds directly to an equal reduction in the number of STV-only infected cells due to co-infection by DIPs. This reasoning was exploited by B&C to relate the STV yield reduction to the reduction in the number of STV-only infected cells, i.e. cells that received one or more STV but received no DIPs, and in turn relating that number to the number of DIPs in the infecting inoculum based on the Poisson distribution, e.g. an infectious virus yield that is 37% of that in the absence of DIPs implies that cells were inoculated at a DIP MOI of 1 DIP per cell.

According to the Poisson distribution, on which B&C's reasoning relies, the fraction of STV-only infected cells, and thus the STV yield, should decrease exponentially as the number of DIPs in the inoculum is increased. When expressing the relative STV yield on a logarithmic scale versus the linear increase in dose of DIPs received, as in figure 3*a*, the data points should fall on a straight line with a slope equal to the DIP MOI (DIP per cell) of the undiluted sample. Thus, having performed the B&C assay to produce the graph shown in figure 3*a*, the DIP MOI of a sample can be determined either directly from the slope of this graph or by taking the reciprocal of the sample dilution factor at which the relative infectious virus yield is 37%, e.g. in figure 3*a*, the reciprocal of 25% or 1/4 = 4 DIPs per cell.

**Figure 4.**
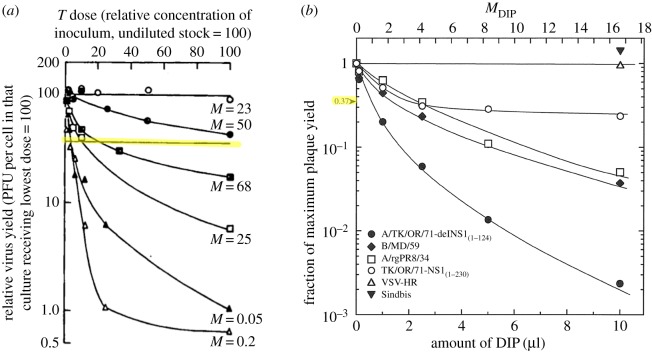
Experimental deviation from the theoretical predictions of B&C. Data do not follow the exponential relationship (which should appear as a straight line on these graphs) theoretically predicted by B&C, though this deviation is due to different factors in each case. (*a*) B&C assay applied to VSV where the lines correspond to different STV MOI of the stock DIP-free virus. (Reproduced with added highlighting from [20] (Copyright 1959, Microbiology Society).) (*b*) B&C assay applied to quantify DIPs in an influenza A virus sample using different DIP-free virus stocks. (Reproduced with permission from [21] (Copyright 2009, American Society for Microbiology).) Despite this, the authors estimate the DIP MOI of the samples using the reciprocal of the sample concentration corresponding to a 37% relative infectious virus yield (highlighted).

However, even in B&C's original publication, the same assay was performed using different DIP-containing VSV samples, in conjunction with varying STV MOIs, and yielded data that did not follow a straight line on a logarithmic-linear plot, as shown in figure 4*a*. Although the data clearly disagreed with their theoretical prediction, the authors calculated the undiluted samples' DIP MOI from the reciprocal of the sample dilution factor at 37% relative STV yield. More recently, Marcus *et al*. used the B&C assay to count DIPs in different influenza A virus samples [21]. Again, despite marked deviations of their data from the theoretically predicted trend, Marcus *et al*. calculated the sample's DIP MOI from the reciprocal of the sample dilution factor corresponding to a 37% relative STV yield, as shown in figure 4*b*. If the experimental data obtained by performing the B&C assay deviate from the theoretically predicted behaviour, is the DIP MOI they yield still accurate? Answering this question requires an understanding of the conditions that give rise to these deviations so as to either avoid them or modify B&C's assumptions and expand their theoretical framework to account for them.

### 2.2. A mathematical model of influenza A STV and DIP infection kinetics

**Figure 5.**
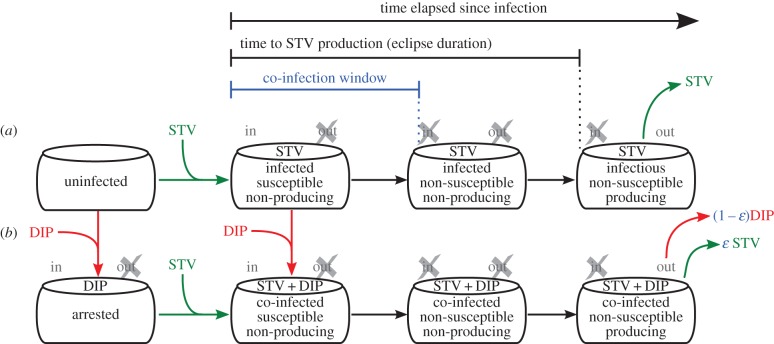
Mathematical model for the kinetics of influenza A virus infection in the presence of DIPs. (*a*) The standard branch of infection depicts the progression of an STV-infected cell through an eclipse phase (infected but not yet producing STV) followed by an infectious phase (infected and STV-producing). (*b*) The co-infection branch shows a DIP-infected cell in an arrested state, until co-infection by STV, which triggers its progression through the eclipse phase, and infectious phase wherein the DIP + STV co-infected cell can produce DIPs, but possibly also STV progeny. Two key aspects of DIP co-infection are highlighted (blue): the fraction of progeny STV produced by co-infected cells (*ɛ*), and the co-infection window, i.e. the time after entering the eclipse phase at which an STV-infected cell ceases to be susceptible to further infection.

The calculation behind the B&C assay stems from a static view of infection; it overlooks the kinetics of virus attachment, replication and release, and how these affect a cell's susceptibility to re-infection. To explore these aspects of infection and determine how they can manifest in a B&C assay, we employed a mathematical model for influenza A STV and DIPs, with emphasis on the details of co-infection. The mathematical model, illustrated in figure 5, takes a mesoscopic view of infection, describing only quantities of particles and cells in various states. Hereafter, we will refer to this new mathematical model as the LIB (Liao, Iwami and Beauchemin) model.

In the LIB model, uninfected cells can be infected by STV. Cells newly infected by STV are not yet able to produce STV for a length of time called the eclipse phase. At the end of the eclipse phase, STV-infected cells produce progeny STV for a length of time called the infectious phase, until the cells die. Uninfected cells can also be infected by DIPs, and enter an arrested state. Arrested cells remain in that state forever, or until they are co-infected by STV. Herein, the term co-infected cell will be used to refer only to a cell that has been infected by no less than one STV and one DIP, but does not refer to cells super-infected by more than one STV and no DIPs. Like STV-infected cells, cells co-infected with DIP + STV proceed through an eclipse phase during which they produce no STV, and after which they will produce progeny for some time until cell death. The mathematical details of the LIB model are provided in the electronic supplementary material, section S5.

The standard, DIP-free branch of the LIB model (figure 5*a*) has been used and extensively validated for influenza A virus infections [11,27–29]. In order to explicitly capture influenza A virus infection kinetics in the presence of DIPs, the model was expanded (figure 5*b*) to incorporate two important aspects of DIP co-infection: the co-infection window, and the fraction of progeny STV produced by a co-infected cell. The co-infection window is defined as the duration post-infection for which a newly influenza A STV-infected cell remains susceptible to co-infection by DIPs. The LIB model allows for the co-infection window to vary in length from 0 h up to, and not exceeding, the length of the eclipse phase. In principle, the co-infection window could extend beyond the end of the eclipse phase, i.e. beyond the time at which a cell has begun producing and releasing STV. This is not the case for infection with influenza A STV, as explained in the electronic supplementary material, section S1.

The co-infection window introduces a second route to co-infection, i.e. STV-first, followed by DIP. It is important to note that the co-infection window in the LIB model is only defined for co-infection by STV-then-DIP, and not DIP-then-STV. In the case of the latter, the LIB model assumes that DIP-only infected cells will remain in the arrested state forever (infinitely long co-infection window), until they are co-infected by STV. This asymmetry arises because the LIB model is constructed with influenza A DIPs in mind, and assumes DIPs contain a large deletion in one of the vRNA segments that encode viral polymerase. If the LIB model considered DIPs containing DI vRNAs of other gene segments, the timing of co-infection with DIP-then-STV could require its own co-infection window, like that for STV-then-DIP. For example, if the defect in DIPs was such that their replication could proceed to shutdown vRNA synthesis, delayed co-infection by DIP-then-STV could abolish production of both progeny, as was explored via a mathematical model in [30].

The other important aspect of DIP co-infection is the fraction of progeny STV produced by co-infected cells. In the LIB model, this is controlled by a fraction *ɛ* which can take a value between 0 (co-infected cells produce only DIPs) and 1 (co-infected cells produce only STV). The LIB model assumes *ɛ* is independent of the number of infecting STV and DIP. However, such a dependence might be warranted if the number of infecting particles could reverse DIP-mediated interference for influenza A virus, as suggested in [31]. In electronic supplementary material, section S2, we explain why the experimental results in [31] do not provide definitive evidence for the reversal of DIP interference, hence this process is neglected in the LIB model. Throughout our work below, co-infected cells are assumed to exclusively produce DIPs (*ɛ* = 0), unless otherwise stated.

Finally, because the duration of incubation prior to rinsing of the inoculum, the rate of infection, virus diffusion, affinity for cells, etc., are all factors that affect the measured infectivity of an STV or DIP in a sample, infectivity is an inherently relative quantitative measure. For the purpose of the present work, when we refer to a sample as containing 0.01 PFU per cell, we mean one that results in the infection of 1% of cells *in the context of that experiment*, given the virus infection rate or affinity, its rate of loss of infectivity and the incubation time if the inoculum is rinsed. Similarly, we describe a sample as containing 0.01 DIPs per cell if it results in 1% of cells becoming DIP-only or DIP + STV infected *in the context of that same experiment*.

### 2.3. Effect of the co-infection window

**Figure 6.**
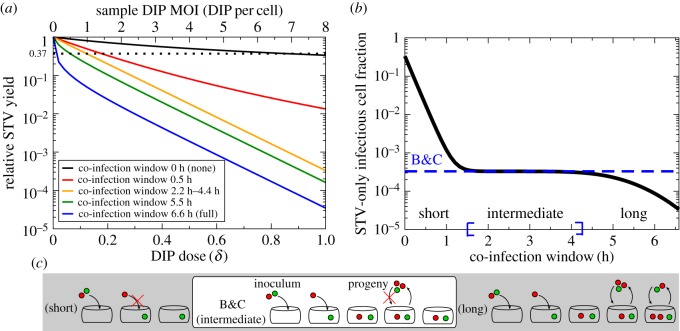
Impact of the co-infection window in the B&C assay. (*a*) Simulated B&C assay using the LIB model for cells infected with an inoculum of DIP-free influenza A STV stock at 4 PFU per cell and varying dilutions of an example sample containing 8 DIPs per cell when undiluted. The various lines correspond to various co-infection window lengths. (*b*) The B&C estimate for the fraction of STV-only infectious cells (dashed line) compared against the actual number predicted by the LIB model (solid line) for various co-infection window lengths (inoculum = 4 PFU per cell + 8 DIP per cell). (*c*) For a short co-infection window, cells that receive at least one PFU will immediately exclude co-infection by DIPs. For a long co-infection window, cells remain susceptible to co-infection long enough to be co-infected by DIP progeny rather than only by the initial inoculum. For an intermediate window, the Poisson distribution on which B&C rely correctly accounts for the relationship between the initial infecting inoculum and STV yield. The B&C estimate is valid for co-infection windows from 1.5 h to 4.3 h given an eclipse phase of 6.6 h.

In figure 6*a*, the LIB model was used to simulate the B&C assay by infecting cells with an inoculum of DIP-free STV stock at 4 PFU per cell plus varying dilutions of an example sample at 8 DIPs per cell when undiluted. The co-infection window length is varied from 0 h (i.e. a cell infected by STV first cannot be co-infected by DIP) to the length of the eclipse phase, 6.6 h in this example (i.e. a cell infected by STV first remains susceptible to co-infection by DIP right up until it starts to produce and release STV). As the length of the co-infection window is increased, the slope of the simulated data increases, causing the estimate for the number of DIPs in the example sample based on the B&C calculation to increase. Therefore, the length of the co-infection window relative to the eclipse length has an important impact on the DIP MOI estimated using the B&C assay.

To better understand how the co-infection window can cause deviations from the theoretically predicted trend in the B&C assay, the LIB model was used to simulate an influenza A virus infection with inoculum MOIs of 4 PFU per cell + 8 DIPs per cell (figure 6*b*). The Poisson distribution—exploited by B&C to relate inoculum content to the fraction of STV-only infected cells—predicts the fraction of STV-only infected cells, given the inoculum, to be 3.29 × 10^−4^, with no consideration or accounting for the length of the co-infection window. At intermediate co-infection window lengths—1.5 h–4.3 h for a 6.6 h eclipse length—the B&C assay provides an accurate count of the inoculum DIP MOI. However, when the co-infection window is either very short or very long relative to the eclipse phase, B&C's prediction based on the Poisson distribution disagrees with the fraction of STV-only infected cells simulated with the LIB model, and yields an incorrect estimate of the inoculum DIP MOI.

The two separate processes responsible for the disagreement for very short and long co-infection windows are depicted in figure 6*c*. With the shortest co-infection window (0 h), infection with DIP-then-STV will result in co-infection, but infection with STV-then-DIP, is prevented. In their use of the Poisson distribution, B&C assume that the order of infection (DIP followed by STV or STV followed by DIP) is not important. While it is easy to account for and correct this theoretically for a co-infection window of 0 h, it becomes increasingly complicated for non-zero co-infection window lengths because the timing of the DIP infection after STV infection becomes relevant. As shorter co-infection windows lead to fewer co-infected cells, and more STV-only infected cells producing greater STV yields, the use of the B&C assay will result in an *underestimation* of the true DIP MOI. On the other hand, when the co-infection window is almost as long as the eclipse phase, the first cells to be infected by the inoculum will begin producing progeny while the last cells to be infected remain susceptible to co-infection such that a number of these cells will be co-infected by the DIP progeny of cells infected early. B&C account only for infection by the initial inoculum, and not for secondary infection from the progeny. Long co-infection windows will result in more co-infected cells, fewer STV-only infected cells, and therefore less STV yields than predicted by B&C. As such, use of B&C with viruses that have a long co-infection window will result in an *overestimation* of the true DIP MOI of the sample.

### 2.4. Length of an influenza A virus co-infection window

Given this restriction on the validity of the B&C method, i.e. that the co-infection window is of intermediate length relative to the eclipse phase, it is necessary to determine a STV's co-infection window length prior to using the B&C assay to ensure the latter will yield a correct estimate. In 1978, Nayak *et al*. performed an experiment with influenza A STV and DIPs, which provides a good estimate of the co-infection window length [12]. Cell cultures were inoculated with a DIP-free, influenza A STV stock (1 PFU per cell, adsorbed for 30 min), and at various times prior to, concurrent with, or after, were inoculated again with a sample containing a high MOI of DIPs (approx. 0.004 PFU per cell + 4 DIPs per cell, adsorbed for 30 min). At 14 h after inoculation with the DIP-free STV stock, the supernatant was harvested for subsequent quantification of the STV yield.

**Figure 7.**
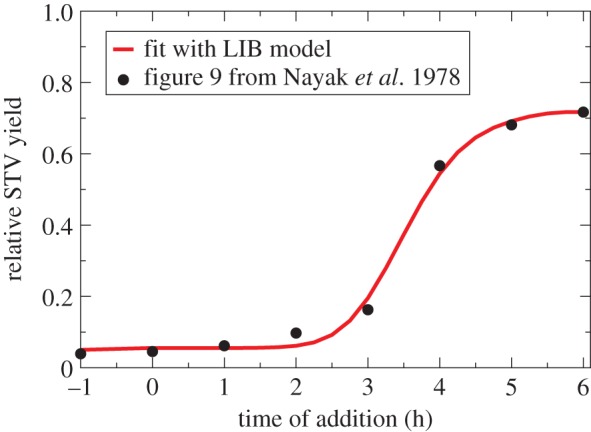
The length of the co-infection window for influenza A virus. Cells were infected with a DIP-free, influenza A STV stock at approximately 1 PFU per cell, followed by the addition of a DIP-containing sample at approximately 2 DIPs per cell at various times post-infection (*x*-axis). The STV yield is sampled 14 h after infection with the STV stock, and expressed relative to the STV yield from an STV stock-only (DIP-free) infection (*y*-axis). The best fit of the LIB model to the data taken from figure 9 in Nayak *et al*. [12] identifies a 3.5 h co-infection window, relative to a 7.1 h eclipse length (for fitting details, see electronic supplementary material, section S5).

In order to identify the co-infection window for influenza A STV from the data generated by Nayak *et al*., the LIB model was fitted to the dataset, as shown in figure 7. The fitting procedure identified a 3.5 h co-infection window, which is an intermediate length relative to the eclipse length, estimated as 7.1 h based on these data. Hence, the co-infection window of this influenza A STV strain makes it suitable for use of the B&C assay to obtain accurate estimates of the DIP MOI. This result is discussed further in electronic supplementary material, section S1. To obtain a more accurate estimate for the co-infection window, this experiment should be performed using a higher MOI of DIP-free STV inoculum at 4 PFU per cell (see electronic supplementary material, section S5).

### 2.5. Effect of allowing co-infected cells to produce progeny STV

**Figure 8.**
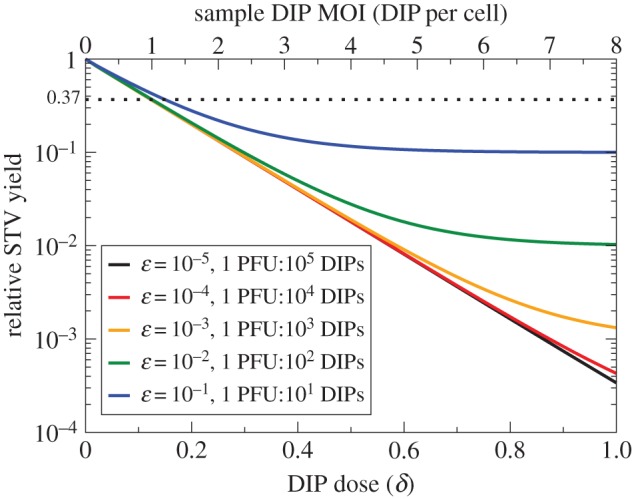
Impact of non-negligible STV yield by co-infected cells in the B&C assay. Simulated B&C assay using the LIB model for cells infected with an inoculum of DIP-free influenza A STV stock at 4 PFU per cell and varying dilutions of an example sample containing 8 DIPs per cell when undiluted. The various lines correspond to various fractions, *ɛ*, of STV per DIP produced by co-infected cells. For STV production by co-infected cells below 1 PFU per 1000 DIPs (*ɛ* = 10^−3^), the deviation from the theoretically predicted trend for the B&C curve is relatively small, i.e. it probably would not be statistically significant given the typical experimental uncertainty in STV yield measurements, and should yield reasonably accurate estimates for the DIP MOI of a sample.

The calculation behind the B&C assay relies on the assumption that cells co-infected with DIP + STV will produce only DIPs, and no STV progeny. In figure 8, the B&C STV yield reduction assay is simulated using the LIB model as the fraction of STV progeny produced by co-infected cells, *ɛ*, is varied from 10^−5^ to 0.1 STV (PFU) produced per DIP, or 10 to 100 000 DIPs produced per STV (PFU).

With more STV progeny produced per DIP by co-infected cells (larger *ɛ*), the B&C curve bends upwards and a saturation in the STV yield reduction is observed. Saturation is attained when the DIP dose is sufficient to co-infect all cells, and the saturation value is equal to the fraction of STV produced per DIP by co-infected cells, *ɛ*. With fewer STV progeny produced by co-infected cells (smaller *ɛ*), the B&C curve is more consistent with its theoretically predicted/assumed linear trend (i.e. no saturation). As experimental variability in measurements of STV yield is typically greater than half an order of magnitude, the production of less than 10^−3^ PFU per DIP by co-infected cells would probably yield a B&C curve that is statistically indistinguishable from, i.e. looks consistent with, the theoretically predicted trend which assumes no STV yield by co-infected cells. Thus, use of the B&C assay—and its assumption that co-infected cells produce no STV progeny—should provide a reasonable estimate of the DIP MOI of a sample as long as co-infected cells produce no more than 1 PFU per 1000 progeny DIPs produced.

### 2.6. STV production by cells co-infected by influenza A STV and DIP

**Figure 9.**
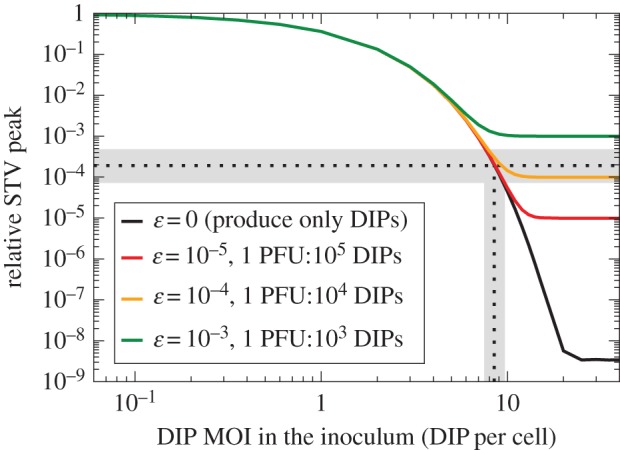
STV produced by cells co-infected with influenza A STV and DIPs. The simulated relative STV peak (*y*-axis) as a function of the possible, unknown DIP MOI in the 2009 influenza A H1N1 pandemic virus sample (*x*-axis). The various lines correspond to various fractions of STV produced by DIP + STV co-infected cells. The experimentally observed (figure 2) relative STV peak of 2 × 10^−4^ is indicated (horizontal dashed line) along with its 95% CI (horizontal grey bar). Cells co-infected with this 2009 influenza A pandemic STV + DIP would have to produce no more than 1 PFU per 10 000 DIPs (*ɛ* ≤ 10^−4^) in order to reproduce the relative STV peak observed experimentally, and the MOI of DIPs in the inoculum was likely approximately 8.5 DIPs per cell (vertical dashed line indicating where the curves cross the horizontal dashed line).

In figure 2, the presence of DIPs in a high MOI infection with a 2009 influenza A H1N1 pandemic virus strain was shown to decrease peak STV titre by 5000-fold relative to an infection inoculated at low MOI. To reproduce the effect seen in this experiment, two infections were simulated with the LIB model: one receiving 4 PFU per cell and no DIPs, the other receiving 4 PFU per cell and an unknown DIP MOI. The STV peak from the DIP-containing infection relative to that of the DIP-free infection was computed and compared with the 2 × 10^−4^ (1/5000) relative peak drop observed with the 2009 influenza A H1N1 pandemic virus strain sample. In figure 9, this relative STV peak is shown as a function of the possible DIP MOI in the sample, under various assumptions regarding the fraction of STV progeny produced by co-infected cells.

When co-infected cells are assumed to exclusively produce DIPs, an initial inoculum containing 8.5 (7.5 DIPs per cell–9.7 DIPs per cell) is required to match the experimentally observed relative STV peak. Given that the inoculum contained 4 PFU per cell, the experimental sample of 2009 influenza A pandemic virus used to inoculate the infections shown in figure 2 probably contained 1.88 DIPs per PFU–2.43 DIPs per PFU, or about twice as many DIPs than PFU.

If co-infected cells do indeed produce some STV progeny, they would have to produce less than 1 PFU for every 10^4^ progeny DIPs (*ɛ* ≤ 10^−4^) in order to reproduce the observed relative STV peak. Hence, cells co-infected with this influenza A STV + DIP could not produce more than 1 PFU for every 10^4^ progeny DIPs, a level of STV production that is negligible in the context of the B&C assay, as shown in figure 8. Thus, use of the B&C assay should be appropriate to estimate the DIP MOI for this influenza A virus strain (A/Québec/144147/09 (H1N1)) because it produces less than the 1 PFU per 1000 progeny DIPs required for the B&C assay to remain accurate. This result is discussed further in electronic supplementary material, section S1.

### 2.7. Effect of using STV stock at low MOI to perform the B&C assay

Herein, thus far, the following requirements have been identified to ensure the B&C assay is suitable to accurately quantify DIPs: (i) a co-infection window of intermediate length relative to the eclipse phase and (ii) cells co-infected with DIP + STV must produce fewer than 1 PFU for every 1000 progeny DIPs. Our analysis of experiments performed with various influenza A viruses suggests that this virus does appear to meet these requirements. Yet, in figure 4, important deviations from the theoretically predicted linear trend can be observed for the B&C assay performed by Marcus *et al*. to quantify influenza A DIPs [21]. Thus, there must be additional criteria to be met in order for the B&C assay to function properly. In [21], Marcus *et al*. cite the findings of Akkina *et al*. [31]—namely, the reversal of DIP-mediated interference at high STV MOIs (see electronic supplementary material, section S2)—to justify conducting their B&C assay with a low MOI of 0.3 PFU per cell, rather than a high MOI, of their relatively DIP-free STV stock (obtained by passaging at low MOI). Unfortunately, such a low STV MOI leaves 74% (e^−0.3^ × 100%) of cells uninfected by STV, and thus remaining susceptible to infection by the DIP and STV progeny from a second cycle of infection. As discussed above, the B&C calculation relies exclusively on the content of the inoculum and does not account for infections resulting from a second cycle of infection.

**Figure 10.**
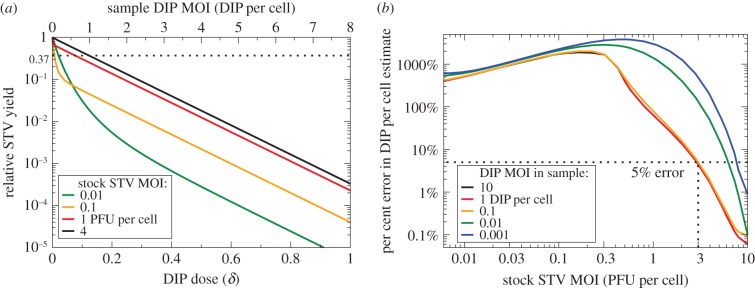
Impact of the STV MOI of the DIP-free STV stock. (*a*) Simulated B&C assay for inoculation with serial dilutions of an example sample containing 8 DIPs per cell when undiluted, plus a DIP-free STV stock. The various lines correspond to various MOIs of the DIP-free STV stock used in conducting the assay. (*b*) The relative per cent error in the DIP MOI estimated using the B&C assay—when computed from the reciprocal of the sample dilution at 37% relative STV yield—as a function of the DIP-free STV stock MOI used. The various lines correspond to the actual DIP MOIs in the undiluted sample being evaluated through the B&C assay. The B&C assay is most accurate (error less than 5%) for high DIP sample (more than 0.1 DIPs per cell) and STV stock (more than 3 PFU per cell) MOIs.

In figure 10*a*, the B&C assay is simulated for infections inoculated with varying MOIs of the DIP-free STV stock plus a series of dilutions of an example sample, which contains 8 DIPs per cell when undiluted. The simulations assume a 3 h co-infection window, relative to the 6.6 h eclipse length, and assume that co-infected cells exclusively produce DIPs, i.e. produce no STV (*ɛ* = 0). As the STV MOI in the DIP-free STV stock is decreased, the simulated B&C assay deviates from the theoretically predicted linear trend at low DIP doses, i.e. at high dilutions of the DIP-containing sample being measured. The desired linear trend, yielding a correct DIP count, is obtained when using a DIP-free STV stock of 4 PFU per cell, but not for STV MOIs at, or below, 1 PFU per cell. When using a DIP-free STV stock at MOIs that are too low, the B&C assay *always overestimates* the DIP MOI in the sample. As shown in figure 10*b*, at an STV stock MOI of 2.5 PFU per cell used by B&C [20], the B&C assay overestimates the DIP MOI by approximately 10%, or 1.1-fold, an error that is probably smaller than the experimental uncertainty expected in an actual experiment. At the STV MOI of 0.3 PFU per cell used by Marcus *et al*. [21], the overestimate would be no less than 1600%, or at least 17 times larger than the true DIP MOI in the sample. Generally, when using the B&C assay to estimate the MOI of DIPs in a sample containing a high MOI of DIPs (at least more than 0.1 DIPs per cell undiluted), a DIP-free STV stock MOI of no less than 3 PFU per cell should be used to keep the error in the estimate below 5%. Otherwise, if the sample contains a low MOI of DIPs (less than 0.1 DIPs per cell undiluted), then an even higher DIP-free STV stock MOI is needed to keep the error in the estimate around 5%, as shown in figure 10*b*.

The causes of curvature are summarized in electronic supplementary material, section S4, which includes an explanation of the B&C assay's sensitivity to the DIP MOI of the sample (electronic supplementary material, section S3).

## 3. Discussion

In 1959, B&C developed an *in vitro* assay to quantify DIPs. The calculations underlying B&C's method dictate that the assay curve be a decreasing straight line, i.e. there should be a linear relationship between the logarithm of the quantity with which DIPs interfere (B&C used the infectious STV yield) as a function of increasing DIP dose. From this linear relationship, the DIP MOI of an unknown sample can be estimated. In many publications making use of interference assays based on the B&C assay, the assay data do not follow the requisite linear trend [16,20,21,24–26], yet the DIP MOI was still estimated using the B&C calculations. The deviation of the assay data from the linear relationship has never been fully explained. We asked whether the B&C assay could still correctly estimate the DIP MOI when these deviations are apparent, and if not, we were further interested in determining if there were any constraints on the applicability of the B&C assay that were not stated in the original publication [20].

We used a mathematical model of influenza A virus infection (LIB model) to simulate *in vitro* infections in the presence of DIPs, in order to reproduce and verify the B&C assay. In the LIB model, we introduced two key co-infection parameters: the co-infection window (how long after STV infection cells block further infection by other virions), and the fraction of STV to DIP progeny (PFU per DIP) produced by cells co-infected by DIP and STV. In general, our results show that the B&C assay will correctly estimate the DIP MOI in a sample when: (i) an STV-infected cell's co-infection window is approximately half the length of its eclipse phase (how long after initial infection the cell begins to produce progeny virion), (ii) cells co-infected by STV and DIP produce fewer than 1 STV per 10^3^ progeny DIP and (iii) the B&C assay is performed using a high STV MOI (greater than 4 PFU per cell) for initial infection. To our knowledge, this is the first time explicit conditions for proper use of the B&C assay have been identified.

In order to ensure the DIP MOI of a sample can be reliably estimated by performing a B&C assay, one should visually verify that the data generated by their B&C assay follows a straight line, as described in more detail herein. If this is not the case then the curvature could be due to

*Cause 1:* the use of a DIP-free STV stock which infects cells with an STV MOI that is too low. The STV stock should be concentrated until a high STV MOI can be achieved;
*Cause 2:* too high a DIP MOI in the sample whose DIP content is to be quantified. The sample should be diluted further before it is used to perform the B&C assay;
*Cause 3:* the key co-infection parameters of the specific virus of interest (i.e. length of co-infection window, fraction of STV produced by co-infected cells) not meeting the conditions we outline herein; and/or
*Cause 4:* the specific virus of interest having a mechanism of interference inconsistent with that assumed by the B&C assay and described herein.

While the first two causes can be addressed by modifying the experimental procedure, the last two are virus-dependent and thus cannot be remediated. In such a case, a different assay would need to be designed and our findings might not apply.

Throughout our work, we made several reasonable assumptions, and so, our results should be taken with the following caveats in mind. First, we assumed that the STV stock was DIP-free. Practically, we do not know how close to ‘DIP-free’ an STV stock can be made, because the effectiveness of methods to reduce the concentration of DIPs (e.g. low MOI passaging) remains yet to be quantified. However, we expect that an STV stock containing DIPs would only diminish the B&C assay's sensitivity to samples containing very high DIP concentrations, and this can be easily fixed by further diluting the sample whose DIP content is to be quantified using the B&C assay. Second, we assumed that the generation of de novo DIPs by STV-only infected cells was a negligible source of DIPs, compared with the amplification of DIPs already in a sample. This is reasonable because replicase errors should occur relatively infrequently. Even if the generation of DIPs was not negligible, the LIB model predicts there would be no impact on the relative yield if the B&C assay is performed properly as a single-cycle infection, with an intermediate co-infection window and low fraction of STV progeny produced by co-infected cells. As such, the findings presented herein hold irrespective of these assumptions. Additionally, our validation of B&C is linked to the assumptions made in the LIB model specific to the mechanisms of interference by influenza A DIPs. These include the assumption that DIP-only infected cells remain susceptible to STV co-infection for any length of time; that the timing of DIP + STV co-infection does not affect the amount or ratio of progeny produced; that the magnitude of the interference is independent of the number of infecting STV and DIP; and that both infected and co-infected cells produce the same amount of progeny. Herein, we provide justification for these assumptions in the context of influenza A STV and DIP infection. If a virus' mechanism for interference is different from that assumed here, then there could yet be other reasons why that virus cannot be characterized by B&C. For example, DIPs of VSV have been shown to exhibit a multiple-hit inhibition mechanism, which is incompatible with both the LIB model and the assumptions of the B&C assay, as shown in [32,33].

To our knowledge, all assays to quantify the presence of DIPs in a sample to date rely on the same principles and calculations as the B&C assay, differing only in their endpoint, i.e. the *y*-axis in the B&C plot. B&C assume that DIP + STV co-infected cells produce only DIP such that the reduction in STV yield (their endpoint) corresponds to a proportional reduction in STV-only infected cells in favour of STV + DIP co-infected cells, as performed in [20–22,33,34]. In the variation of the B&C assay performed in [24,35,36], after inoculation and incubation, infected and co-infected cells are trypsinized, and plated onto a new monolayer under agar. In their assay, the reduction in STV-only infected cells is counted as a reduction in visible plaques, rather than a reduction in STV yield. As such, this assay is in all ways equivalent to that performed by B&C, and our findings apply to it also. The other variation of the B&C assay performed in [16,23,25,26,37] assumes that cells co-infected by STV + DIP will be protected from cytopathic effects (CPE) whereas those infected by STV alone will exhibit CPE. In their assay, the reduction in STV-only infected cells is counted as a reduction in CPE, rather than a reduction in STV yield. Herein, because of our focus on influenza A virus, we have assumed both STV-only and STV + DIP-infected cells will have the same lifespan and thus exhibit the same CPE profile. This assumption is supported by both our prior work [11,27–29] and findings reported in [21], but is in contradiction with that reported in [37], all in the context of influenza A virus infections in the presence of DIPs. Nonetheless, for viruses where DIP co-infection is truly protective from CPE, use of the CPE as the endpoint in the B&C calculation will result in a valid, linear relationship only if the virus' co-infection window is of intermediate duration relative to its eclipse phase (as with STV yield reduction), but would not require that co-infected cells produce negligible amounts of STV (as is needed when using STV yield reduction). As we did not explore such a mechanism herein, however, it is possible that using CPE as the endpoint requires additional constraints not explored here.

While our work explains why not obtaining a linear relationship means B&C is not valid, we cannot guarantee that obtaining a straight line means B&C is valid. As such, it is important to ensure the above criteria are met even when a linear trend is obtained. The B&C assay has already been applied to viruses such as the Sindbis virus [34,35], lymphocytic choriomeningitis virus [23–25], infectious pancreatic necrosis virus [16], respiratory syncytial virus [26], mumps virus [22] and influenza A virus [21,36,37]. Herein, we describe reliable procedures to determine a virus' key co-infection parameters and verify Cause 3 via simple, conventional, *in vitro* infection experiments, using influenza A virus as an example, so that others can follow our methods with their virus of interest. We hope that our work, and that of others before us [32,33], will motivate others to do so. When applied correctly to quantify the DIP content of a suitable virus, the B&C assay is an effective methodology to reliably monitor DIP accumulation in high yield processes, determine actual dosages when DIPs are used as antivirals, and resolve the extent to which existing methods to reduce DIP contents in virus samples are effective. For viruses that do not meet the criteria identified herein, our LIB model, with appropriate modifications to capture the specific interference mechanism of that virus, could potentially help test alternatives to the B&C assay or even be used to directly quantify DIPs in that virus sample.

## Supplementary Material

Supplementary Material

## References

[1] Carter MJ, Mahy BWJ 1981 Incomplete avian influenza virus contains a defective non-interfering component. Arch. Virol. 71, 13–25. (10.1007/BF01315172) 7065901

[2] Huang AS, Baltimore D 1970 Defective viral particles and viral disease processes. Nature 226, 325–327. (10.1038/226325a0) 5439728

[3] Dimmock NJ, Easton AJ 2014 Defective interfering influenza virus RNAs: time to reevaluate their clinical potential as broad-spectrum antivirals? J. Virol. 88, 5217–5227. (10.1128/JVI.03193-13) 24574404 PMC4019098

[4] Davis AR, Hiti AL, Nayak DP 1980 Influenza defective interfering viral RNA is formed by internal deletion of genomic RNA. Proc. Natl Acad. Sci. USA 77, 215–219. (10.1073/pnas.77.1.215) 6928614 PMC348239

[5] Davis AR, Nayak DP 1979 Sequence relationships among defective interfering influenza viral RNAs. Proc. Natl Acad. Sci. USA 76, 3092–3096. (10.1073/pnas.76.7.3092) 290988 PMC383769

[6] Jennings PA, Finch JT, Winter G, Robertson JS 1983 Does the higher order structure of the influenza virus ribonucleoprotein guide sequence rearrangements in influenza viral RNA? Cell 34, 619–627. (10.1016/0092-8674(83)90394-X) 6616623

[7] Marriott AC, Dimmock NJ 2010 Defective interfering viruses and their potential as antiviral agents. Rev. Med. Virol. 20, 51–62. (10.1002/rmv.641) 20041441

[8] Frensing T 2015 Defective interfering viruses and their impact on vaccines and viral vectors. Biotechnol. J. 10, 681–689. (10.1002/biot.201400429) 25728309

[9] Duhaut SD, McCauley JW 1996 Defective RNAs inhibit the assembly of influenza virus genome segments in a segment-specific manner. Virology 216, 326–337. (10.1006/viro.1996.0068) 8607262

[10] Odagiri T, Tashiro M 1997 Segment-specific noncoding sequences of the influenza virus genome RNA are involved in the specific competition between defective interfering RNA and its progenitor RNA segment at the virion assembly step. J. Virol. 71, 2138–2145. 9032347 10.1128/jvi.71.3.2138-2145.1997 PMC191316

[11] Pinilla LT, Holder BP, Abed Y, Boivin G, Beauchemin CAA 2012 The H275Y neuraminidase mutation of the pandemic A/H1N1 virus lengthens the eclipse phase and reduces viral output of infected cells, potentially compromising fitness in ferrets. J. Virol. 86, 10 651–10 660. (10.1128/JVI.07244-11) PMC3457267 22837199

[12] Nayak DP, Tobita K, Janda J, Davis AR, De BK 1978 Homologous interference mediated by defective interfering influenza virus derived from a temperature-sensitive mutant of influenza virus. J. Virol. 28, 375–386. 702654 10.1128/jvi.28.1.375-386.1978 PMC354277

[13] Kantorovich-Prokudina EN, Semyonova NP, Berezina ON, Zhdanov VM 1980 Gradual changes of influenza virions during passage of undiluted material. J. Gen. Virol. 50, 23–31. (10.1099/0022-1317-50-1-23) 7441212

[14] Frensing T, Pflugmacher A, Bachmann M, Peschel B, Reichl U 2014 Impact of defective interfering particles on virus replication and antiviral host response in cell culture-based influenza vaccine production. Appl. Microbiol. Biotechnol. 98, 8999–9008. (10.1007/s00253-014-5933-y) 25132064

[15] Welsh RM, Pfau CJ 1972 Determinants of defective lymphocytic choriomeningitis interference. J. Gen. Virol. 14, 177–187. (10.1099/0022-1317-14-2-177) 4622135

[16] Macdonald RD, Yamamoto T 1978 Quantitative analysis of defective interfering particles in infectious pancreatic necrosis virus preparations. Arch. Virol. 57, 77–89. (10.1007/BF01315639) 566090

[17] Weiss B, Schlesinger S 1973 Defective interfering passages of Sindbis virus: chemical composition, biological activity, and mode of interference. J. Virol. 12, 862–871. 4798187 10.1128/jvi.12.4.862-871.1973 PMC356705

[18] Huang AS 1973 Defective interfering viruses. Annu. Rev. Microbiol. 27, 101–118. (10.1146/annurev.mi.27.100173.000533) 4356530

[19] Nayak DP 1980 Defective interfering influenza viruses. Annu. Rev. Microbiol. 34, 619–644. (10.1146/annurev.mi.34.100180.003155) 7002033

[20] Bellett AJD, Cooper PD 1959 Some properties of the transmissible interfering component of vesicular stomatitis virus preparations. J. Gen. Microbiol. 21, 498–509. (10.1099/00221287-21-3-498) 13798559

[21] Marcus PI, Ngunjiri JM, Sekellick MJ 2009 Dynamics of biologically active subpopulations of influenza virus: plaque-forming, noninfectious cell-killing, and defective interfering particles. J. Virol. 83, 8122–8130. (10.1128/JVI.02680-08) 19494019 PMC2715774

[22] Šantak M, Markušić M, Balija ML, Kopač SK, Jug R, Örvell C, Tomac J, Forčić D 2015 Accumulation of defective interfering viral particles in only a few passages in Vero cells attenuates mumps virus neurovirulence. Microbes Infect. 17, 228–236. (10.1016/j.micinf.2014.11.006) 25479555

[23] Ziegler CM et al. 2016 The lymphocytic choriomeningitis virus matrix protein PPXY late domain drives the production of defective interfering particles. PLoS Pathog. 12, e1005501 (10.1371/journal.ppat.1005501) 27010636 PMC4806877

[24] Welsh RM, O'Connell CM, Pfau CJ 1972 Properties of defective lymphocytic choriomeningitis virus. J. Gen. Virol. 17, 355–359. (10.1099/0022-1317-17-3-355) 4650728

[25] Popescu M, Schaefer H, Lehmann-Grube F 1976 Homologous interference of lymphocytic choriomeningitis virus: detection and measurement of interference focus-forming units. J. Virol. 20, 1–8. 978787 10.1128/jvi.20.1.1-8.1976 PMC354958

[26] Treuhaft MW, Beem MO 1982 Defective interfering particles of respiratory syncytial virus. Infect. Immun. 37, 439–444. 6288562 10.1128/iai.37.2.439-444.1982 PMC347553

[27] Paradis EG, Pinilla L, Holder BP, Abed Y, Boivin G, Beauchemin CAA 2010 Impact of the H275Y and I223 V mutations in the neuraminidase of the 2009 pandemic influenza virus *in vitro* and evaluating experimental reproducibility. PLoS ONE 10, e0126115 (10.1371/journal.pone.0126115) PMC4439092 25992792

[28] Holder BP, Beauchemin CAA 2011 Exploring the effect of biological delays in kinetic models of influenza within a host or cell culture. BMC Public Health. 11(Suppl 1), S10 (10.1186/1471-2458-11-S1-S10) PMC3317580 21356129

[29] Holder BP, Liao LE, Simon P, Boivin G, Beauchemin CAA 2011 Design considerations in building *in silico* equivalents of common experimental influenza virus assays and the benefits of such an approach. Autoimmunity 44, 282–293. (10.3109/08916934.2011.523267) 21244331

[30] Laske T, Heldt FS, Hoffmann H, Frensing T, Reichl U 2016 Modeling the intracellular replication of influenza A virus in the presence of defective interfering RNAs. Virus. Res. 213, 90–99. (10.1016/j.virusres.2015.11.016) 26592173

[31] Akkina RK, Chambers TM, Nayak DP 1984 Mechanism of interference by defective-interfering particles of influenza virus: differential reduction of intracellular synthesis of specific polymerase proteins. Virus. Res. 1, 687–702. (10.1016/0168-1702(84)90059-5)

[32] Khan SR, Lazzarini RA 1977 The relationship between autointerference and the replication of a defective interfering particle. Virology 77, 189–201. (10.1016/0042-6822(77)90417-2) 190780

[33] Stauffer Thompson KA, Rempala GA, Yin J 2009 Multiple-hit inhibition of infection by defective interfering particles. J. Gen. Virol. 90, 888–899. (10.1128/vir.0.005249-0) 19264636 PMC2889439

[34] Kowal KJ, Stollar V 1980 Differential sensitivity of infectious and defective-interfering particles of sindbis virus to ultraviolet irradiation. Virology 103, 149–157. (10.1016/0042-6822(80)90133-6) 7368573

[35] Johnston RE, Tovell DR, Brown DT, Faulkner P 1975 Interfering passages of Sindbis virus: concomitant appearance of interference, morphological variants, and truncated viral RNA. J. Virol. 16, 951–958. 1165599 10.1128/jvi.16.4.951-958.1975 PMC354757

[36] Janda JM, Davis AR, Nayak DP, De BK 1979 Diversity and generation of defective interfering influenza virus particles. Virology 95, 48–58. (10.1016/0042-6822(79)90400-8) 442544

[37] McLain L, Armstrong SJ, Dimmock NJ 1988 One defective interfering particle per cell prevents influenza virus-mediated cytopathology: an efficient assay system. J. Gen. Virol. 69, 1415–1419. (10.1099/0022-1317-69-6-1415) 3385408

