# Supplementary material for: (In)validating experimentally derived knowledge about influenza A defective interfering particles
Source: J R Soc Interface. 2016 Nov;13(124):20160412. doi: 10.1098/rsif.2016.0412 (PMC5134007; doi:10.1098/rsif.2016.0412)
Supplement: Supplementary Material [file rsif20160412supp1.pdf]

Supplementary Material:
(In)validating experimentally-derived knowledge about influenza A defective interfering particles

Laura E. Liao[1], Shingo Iwami[2,3], Catherine A. A. Beauchemin[1,4,*]

1 Department of Physics, Ryerson University, Toronto, Canada
2 Department of Biology, Kyushu University, Fukuoka, Japan
3 CREST and PRESTO, Japan Science and Technology Agency (JST), Saitama, Japan
4 Interdisciplinary Theoretical Science (iTHES) Research Group at RIKEN, Wako, Japan
***** Corresponding author. Email: `cbeau@ryerson.ca`
Journal of the Royal Society Interface

October 19, 2016

S1 Biological basis of key co-infection parameters of influenza A virus

By simulating a variety of in vitro infection experiments, we determined that influenza A virus infections satisfy the criteria such that the B&C assay will accurately quantify influenza A DIPs when it is properly performed. In the case of influenza A virus, the length of the co-infection window results from either of two mechanisms which block co-infection from happening: superinfection exclusion [1], and/or the shutdown of viral replication [2]. In superinfection exclusion, the appearance of viral neuraminidase (NA) on the cell surface is responsible for the cleavage of sialic acid receptors, preventing the entry of any more particles midway through the viral replication cycle. With the shutdown of viral replication, the M1 viral protein accumulates in the infected cell's nucleus, inhibiting nuclear import of viral RNA, while facilitating its export out of the nucleus to the cell membrane for assembly and packaging. Consequently, any particles which entered during or after the shutdown would be prevented from replicating their vRNA. The co-infection window is likely a combination of particle entry blockage and the halt in replication. We estimated the co-infection window length to be 3.5 h, which we find is consistent with the timing of NA (blocking particle entry) and M1 (replication shutdown). It has been shown that viral neuraminidase begins appearing on the apical surface of STV-infected cells at 1 h–3 h, reaching a maximum at 4 h–7 h post-infection [3,4], while the M1 protein is detectable at 3 h post-infection [2]. It is not necessary in the LIB model to distinguish between either mechanism as the cause of the co-infection window in order to faithfully reproduce viral kinetics in the presence of DIPs.

Our other key co-infection parameter, the fraction of STV progeny produced by a cell co-infected with influenza A STV and DIP, corresponds to the strength of the interference by DIPs. It is believed that DI vRNA has a length advantage over full-length vRNAs, allowing DIPs to competitively inhibit STV replication, but the exact mechanism is unknown [5]. According to one hypothesis generated by a mathematical model [6], DI vRNAs sequester nucleoprotein (NP) to the detriment of full-length vRNAs, since NP is an essential viral protein required for RNA stabilization [7, 8]. Using this mathematical model, Heldt et al. suggest that such a mechanism of interference would result in the almost exclusive production of progeny DIPs from a co-infected cell [9]. Herein, based on analysis of experimental data by the LIB model, we estimate that cells co-infected by influenza A STV and DIPs do not produce more than 1 PFU for every

10^4 progeny DIPs, consistent with both Heldt et al.'s finding, and the general belief that co-infected cells nearly exclusively produce DIPs. In estimating the fraction of STV progeny produced by STV and DIP co-infected cells, we made another interesting finding. Using the LIB model, we determined that the DIP content in our 2009 pandemic H1N1 influenza A STV stock was approximately 2 DIP/PFU. Ordinarily, such an undesirably high DIP concentration in a STV stock would be passaged at low MOI to lower the DIP content [10]. However, our STV stock was made by reverse genetics, and passaging had to be avoided so as not to introduce mutations. When a drop in the peak value of STV is observed (due to DIPs) between multiple-cycle and single-cycle infections, in our experience, we have found *a posteriori* that the virus was made by reverse genetics. For example, peak drops of at least three orders of magnitude were observed in [11–13] for each virus strain generated by reverse genetics.

S2 Alternative explanation for the reversal of DIP-mediated interference

The LIB model assumes that the rate of virion (DIP or STV) production by co-infected cells, and the fraction of that progeny consisting of STV rather than DIPs, is independent of the number of STVs and/or DIPs which co-infected the cell. In other words, the LIB model assumes that a cell infected by one STV and two DIPs will produce virions at the same rate, and the same fraction of its progeny will be STV, as a cell infected by five STV and one DIP.

Challenging this notion, Akkina et al. in 1984, suggest that the fraction of STV vs DIP progeny produced by co-infected cells is dictated by the number of infecting particles, i.e., has a dependence on the ratio of infecting PFU to DIP [14]. Specifically, Akkina et al. propose that the fraction of DIPs produced by co-infected cells is determined by the ratio of full-length, STV vRNAs to DI vRNAs which infect a cell. The idea is that if a cell receives an overwhelming number of STV vRNA copies, this will allow STV vRNA replication to partially overcome the replication advantage held by the shorter-length DI vRNAs. Consequently, according to Akkina et al., further infection of DIP+STV co-infected cells with additional STV should lead to the reversal of the interfering, suppressive effect of DIPs on the STV yield.

Figure S1 (black bars) presents the data which led Akkina et al. to conclude that DIP-mediated interference is reversible [14]. To summarize their data, a drop in STV yield was observed between the DIP-free control (0 DIP/cell + 0.5 PFU/cell) and an infection resulting from inoculation with STV and DIPs (4 DIP/cell + 0.01 PFU/cell). This demonstrates the suppression of STV yields, typical of DIP interference. However, as the STV MOI was increased while keeping the DIP MOI fixed, Akkina et al. claim to observe increasing STV yields. From this, Akkina et al. concluded that increasing the inoculum STV MOI was responsible for reversing the suppressive effect of DIPs and that a greater fraction of STV progeny is produced by co-infected cells when super-infected by STV.

While the data presented in figure S1 are consistent with this hypothesis, they are not conclusive. Figure S1 also presents the results of an experiment simulated with the LIB model (blue bars) demonstrating that, without having to assume that the fraction of progeny made by co-infected cells is influenced by the STV or DIP MOI, it is possible to account for the trend in these data, even under the assumption that co-infected cells strictly produce DIPs. Our alternative explanation of these data is based on the fact that some of the lower STV MOI inocula used by Akkina et al. leave a pool of uninfected cells which are then available to become STV-infected, producing higher STV yields when the inoculum STV MOI is increased. The Poisson distribution predicts that an inoculum containing 4 DIP/cell will infect 98% of cells, leaving 2% of cells free to be infected by any additional STV included in the inoculum. In a typical in vitro infection in 6-well plates where monolayers contain $\sim 10^6$ cells, this would mean $\sim 2 \times 10^4$ cells will remain susceptible to STV. Thus, as the STV MOI in the inoculum is increased, so is the fraction of these cells infected by STV alone, manifesting as the increase in STV yield observed by Akkina et al. Therefore, the DIP portion of the initial inoculum should contain at least 14 DIP/cell, instead of 4 DIP/cell, to ensure that *all* cells are DIP-infected to avoid this effect.

While we can provide an alternative explanation to account for the effect that Akkina et al. claim to have

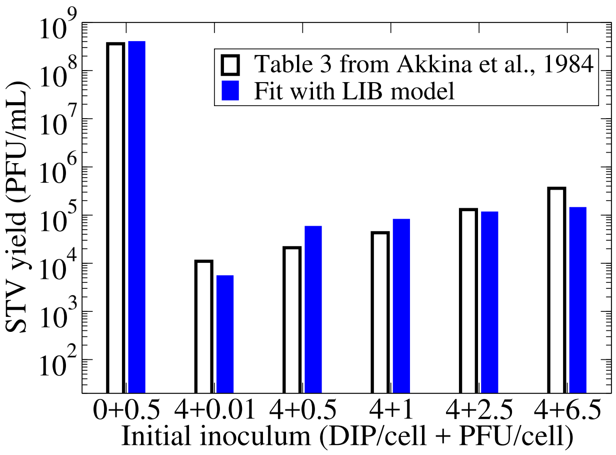

Figure S1: **Evidence by Akkina et al. for a reversal of DIPs' interfering, suppressive effect.** STV yield at 24 hpi for infections initiated with the STV and DIP MOIs indicated (x-axis). A reversal of the STV yield suppression by DIPs appears to occur when a cell is super-infected with additional STV, as per the original dataset by Akkina et al. (black bars, [14]). Using the LIB model (blue bars, see Methods in Section S5), which assumes that co-infected cells will produce the same amount of DIPs and STV, irrespective of the number of STVs or DIPs which infected them, it is possible to reproduce the data by Akkina et al. without the need to assume a reversal of DIP interference.

observed, we also question whether these data even show such an effect at all, i.e., whether the reversal is statistically significant or an irreproducible, statistically significant coincidence. This is made more difficult to ascertain since uncertainties (error bars) were not reported by Akkina et al. in [14]. Some infections were initiated with an inoculum containing a low STV MOI (0.01 PFU/cell, 0.5 PFU/cell, 1 PFU/cell), resulting in multiple cycles of infection. As such, the STV yield observed is a function of not just the composition of the initial inoculum, but also of the progeny resulting from the initial infection. To avoid confounding effects from multiple cycles of infection, all inocula used should have been performed at high MOI (i.e., $>$ 4 PFU/cell) to ensure the effects observed are only due to the inocula. Furthermore, replicate experiments should be performed to determine experimental uncertainty and ascertain the statistical significance of any observed change in yield.

Therefore, the experiment performed by Akkina et al. [14] does not demonstrate, in a definitive manner, the reversal of DIP interference in co-infected cells when these cells are super-infected by additional STV. However, a modified version of their experiment, wherein no inoculum contains less than 14 DIP/cell and no less than 4 PFU/cell, should be sufficient to do so. As more and more STV is added to the inoculum containing 14 DIP/cell, the STV progeny, if any, would be that from co-infected cells only, and their dependence on the number of infecting particles could be assessed, without confounding STV production from a small pool of STV-only infected cells. Such an experiment should be done using multiple replicates to adequately capture experimental variability and establish the statistical significance of any observed effect. It is interesting to note that Heldt et al. have explored the effect of the number of infecting particles on the progeny produced using a mathematical model of a single cell with a detailed representation of intracellular viral replication. They found that a co-infected cell almost exclusively produced DIPs even when there was an increasing number of infecting STV [9]. With the same single cell model [15], Laske et al. matched the results by Akkina et al., however, the reversal of DIP-mediated interference could only recover 1% of the STV yield. Since the LIB model which considers a population of cells, and the model of a single cell by [9,15], can both reproduce these data, this highlights the fact that these data alone are not sufficient to show that reversal of DIP-mediated interference occurs.

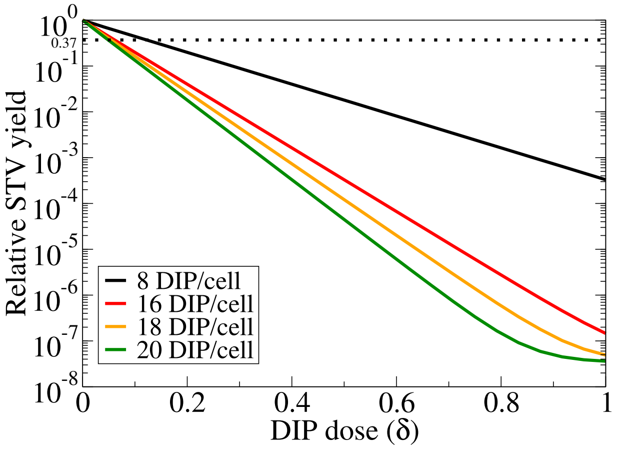

Figure S2: **Impact of the DIP MOI of the sample on the B&C assay.** Simulated B&C assay using the LIB model for cells infected with an inoculum of DIP-free influenza A STV stock at 4 PFU/cell and varying dilutions of an example sample containing either 8, 16, 18, 20 DIP/cell when undiluted.

S3 Sensitivity of the B&C assay to samples containing very high or very low DIP MOIs

In figure S2, the B&C assay is simulated for infections inoculated with the DIP-free STV stock at 4 PFU/cell plus a series of dilutions of example samples containing either 8, 16, 18, or 20 DIP/cell when undiluted. The assay curve for the example samples containing greater than 16 DIP/cell plateaus at high DIP doses and no longer follows a linear trend. The plateau is due to the total suppression of infection even when the sample is diluted such that, any further reduction in yield by additional DIPs beyond 16 DIP/cell is masked by the STV leftover post-rinsing.

At first glance, it appears that the B&C assay is only sensitive to a sample containing up to 16 DIP/cell. However, the sensitivity is determined by the peak value of STV in the infection initiated solely by the DIP-free STV stock (stock-only infection). If the STV yield from the DIP-free infection (at DIP dose of zero) is higher, a greater reduction in yield can be observed, making the assay more sensitive to samples with even higher DIP MOI than 16 DIP/cell. The stock-only infection STV peak is ultimately responsible for the sensitivity of the assay, and depends on the virus-cell kinetics (e.g., virus production rate), or the STV stock purity (i.e., whether the stock is contaminated by DIPs). We can say that due to a STV peak of 10^9 PFU/mL in our simulated stock-only infection, the B&C assay is suitable for counting samples containing up to 16 DIP/cell. When performing the B&C assay with an unknown sample, if a plateau at high DIP dose (low or no dilution of the sample) is observed and other causes of curvature have been ruled out (as summarized in Section S4), a subsequent B&C assay could be repeated with a dilution of the sample at a lower DIP MOI, i.e., in the range where it remains linear.

For samples with very low DIP MOI, the sensitivity of the B&C assay is limited by the experimental variability. Since variability is typically half an order of magnitude, i.e., $\pm 0.5 \log_{10}(\mathrm{TCID}_{50})$ or $\pm 0.5 \log_{10}(\mathrm{PFU})$, we would expect that a B&C assay curve would be indistinguishable from a totally horizontal curve (i.e., insensitive B&C assay) when the relative yield from the undiluted sample (at a DIP dose of 1) is about 0.5. This corresponds to an undiluted sample which contains 0.7 DIP/cell (i.e., $-\log(0.5)$). Thus, the B&C assay is sensitive to samples containing DIP MOIs as low as 0.7 DIP/cell and as high as 16 DIP/cell, depending on the stock-only infection STV peak.

Curvature in the B&C assay

Cause of curvature	At low DIP dose	At high DIP dose
**Property of virus**	Figure 6: Too short/long co-infection window	Figure 8: STV produced by co-infected cells (ε)
**Experimental conditions**	Figure 10: Using too low stock STV MOI	Figure S2: Sample with very high DIP MOI insufficiently diluted

Figure S3: **Classifying types and causes of non-exponential curvature in the B&C assay.** Curvature at low DIP dose occurs either when the co-infection window is too short/long, or when the stock STV MOI is too low. Curvature at high DIP dose occurs either when there is too much STV per DIP produced by co-infected cells, or when the DIP MOI of the sample is too high. The curvature in the B&C assay is either caused by experimental conditions, or stems from an inherent property of the virus.

S4 Classification of types and causes of curvature in the B&C assay

In figure S3, we summarize the two ways in which non-exponential curvature can manifest in the B&C assay: at low DIP doses (high dilution of sample) or at high DIP doses (low dilution of sample). We have already shown in figures 10 and 6 that curvature at low DIP doses is either due to inoculating with a low MOI STV stock (less than 4 PFU/cell), or due to a very long co-infection window, respectively. Likewise, we have shown in figures S2 and 8 that curvature at high DIP dose is either due to a sample containing a very high DIP MOI (greater than 16 PFU/cell, given 10^9 PFU/mL STV peak in the stock-only infection), or due to a large amount of STV progeny being produced by co-infected cells (greater than 1 PFU per 1,000 DIPs), respectively.

In other words, these curvatures can be caused by either a property of the virus (i.e., co-infection window, STV progeny made by co-infected cells) or by the experimental conditions (i.e., STV stock MOI, sample DIP MOI). Of the two causes, it is relatively easy to adjust experimental conditions such as STV and DIP MOIs. Hence, if we believe that the co-infection window and the fraction of STV progeny from co-infected cells are likely not culprits of the B&C assay's non-linear curvature, which is supported by figure 7 and 9, appropriate experimental design should ensure the DIP MOI in an influenza A virus sample can be accurately determined using the B&C assay. Practically, this means that one should perform the B&C assay using a high STV MOI with the unknown DIP sample, and subsequently assess whether the resulting assay curve appears non-linear on a logarithmic-linear plot. If so, the unknown DIP MOI might be too high, and we suggest diluting the DIP sample and repeating the B&C assay until linearity in the assay curve is achieved.

S5 Methods

S5.1 The multiplicity of infection (MOI) determines the fraction of infected cells

It is well-known that the multiplicity of infection (i.e., the ratio of STV in the inoculum to the number of susceptible cells receiving the inoculum) determines the proportion of cells which receive a STV and get infected. Since the process of randomly tossing particles onto an array of cells is exactly described as a Poisson process, we can use the Poisson distribution to mathematically compute the fraction of cells which receive an exact number (N_V) of STV:

$$\text{Fraction of cells receiving } N_V \text{ STV} = \frac{\text{MOI}^{N_V} \exp(-\text{MOI})}{N_V!}$$

To compute the fraction of cells that are STV-infected, we must determine the fraction of cells which received one or more STV, which is equivalent to the total proportion of cells excluding those which did not receive any STV ($N_V = 0$):

$$\text{Fraction of STV-infected cells} = 1 - \frac{\text{MOI}^0 \exp(-\text{MOI})}{0!} = 1 - \exp(-\text{MOI})$$

In mixed infections, when the inoculum contains both STV and DIPs that are distributed randomly and independently onto cells, the fraction of cells that are STV and DIP co-infected is given by

$$\text{Fraction of STV+DIP co-infected cells} = [1 - \exp(-\text{MOI})]\,[1 - \exp(-\text{DIP MOI})]$$

And the remaining fraction of cells in a mixed infection are given by

$$\begin{aligned}
\text{Fraction of STV-only infected cells} &= [1 - \exp(-\text{MOI})]\exp(-\text{DIP MOI}) \\
\text{Fraction of DIP-only infected cells} &= \exp(-\text{MOI})\,[1 - \exp(-\text{DIP MOI})] \\
\text{Fraction of uninfected cells} &= \exp(-\text{MOI})\exp(-\text{DIP MOI})
\end{aligned}$$

S5.2 Mathematical model

The course of in vitro influenza A virus infections in the presence and absence of DIPs was simulated using a mathematical model. The model is based on a simpler one used in previous work wherein it was validated against experimental data for in vitro influenza A virus infections in the absence of DIPs [12, 16]. Since interferon does not have a significant effect on influenza A virus infections in MDCK cells [17], the LIB model does not include a cellular interferon response. To account for the effect of DIPs on infection kinetics, it was modified based, in part, on an earlier model by [18].

$$\begin{aligned}
\frac{\mathrm{d}T}{\mathrm{d}t} &= -\beta T(V+D) & \frac{\mathrm{d}A}{\mathrm{d}t} &= \beta TD - \beta AV \\
\frac{\mathrm{d}E_1}{\mathrm{d}t} &= \beta TV - \frac{n_\mathrm{E}}{\tau_\mathrm{E}}E_1 - z_1\beta E_1 D & \frac{\mathrm{dCE}_1}{\mathrm{d}t} &= \beta AV - \frac{n_\mathrm{E}}{\tau_\mathrm{E}}\mathrm{CE}_1 + z_1\beta E_1 D \\
\frac{\mathrm{d}E_{i=2,\ldots,n_E}}{\mathrm{d}t} &= \frac{n_\mathrm{E}}{\tau_\mathrm{E}}E_{i-1} - \frac{n_\mathrm{E}}{\tau_\mathrm{E}}E_i - z_i\beta E_i D & \frac{\mathrm{dCE}_{i=2,\ldots,n_E}}{\mathrm{d}t} &= \frac{n_\mathrm{E}}{\tau_\mathrm{E}}\mathrm{CE}_{i-1} - \frac{n_\mathrm{E}}{\tau_\mathrm{E}}\mathrm{CE}_i + z_i\beta E_i D \\
\frac{\mathrm{d}I_1}{\mathrm{d}t} &= \frac{n_\mathrm{E}}{\tau_\mathrm{E}}E_{n_\mathrm{E}} - \frac{n_\mathrm{I}}{\tau_\mathrm{I}}I_1 & \frac{\mathrm{dCI}_1}{\mathrm{d}t} &= \frac{n_\mathrm{E}}{\tau_\mathrm{E}}\mathrm{CE}_{n_\mathrm{E}} - \frac{n_\mathrm{I}}{\tau_\mathrm{I}}\mathrm{CI}_1 \\
\frac{\mathrm{d}I_{j=2,\ldots,n_I}}{\mathrm{d}t} &= \frac{n_\mathrm{I}}{\tau_\mathrm{I}}I_{j-1} - \frac{n_\mathrm{I}}{\tau_\mathrm{I}}I_j & \frac{\mathrm{dCI}_{j=2,\ldots,n_I}}{\mathrm{d}t} &= \frac{n_\mathrm{I}}{\tau_\mathrm{I}}\mathrm{CI}_{j-1} - \frac{n_\mathrm{I}}{\tau_\mathrm{I}}\mathrm{CI}_j \\
\frac{\mathrm{d}V}{\mathrm{d}t} &= p\sum_{j=1}^{n_\mathrm{I}} I_j + \varepsilon p\sum_{j=1}^{n_\mathrm{I}} \mathrm{CI}_j - cV & \frac{\mathrm{d}D}{\mathrm{d}t} &= (1-\varepsilon)p\sum_{j=1}^{n_\mathrm{I}} \mathrm{CI}_j - cD
\end{aligned}$$

Equations on the left-hand side correspond to the standard infection model in which uninfected target cells (T) are infected by STV (V) or DIPs (D), at infection rate β. The STV-infected eclipse cells (E) remain infected and non-producing for a time called the eclipse length τ_E, after which they become infectious cells (I), and produce STV over a length of time called the infectious phase τ_I. The STV progeny are produced and released at rate p, and lose infectivity at rate c. The transition times between cell states (eclipse to infectious, infectious to dead) follow a gamma distribution whose mean is given by τ, and standard deviation by $\sigma = \frac{\tau}{\sqrt{n}}$. The parameter n_E (n_I) is the number of compartments the eclipse (infectious) phase is divided into, where the compartments are indexed by i (j). The parameter $z_i = 1$ for $i = 1 : \left(\frac{\tau_C}{\tau_E}\right) n_E$ opens the co-infection window (τ_C) to allow transitions from the standard to co-infected branch, and is zero otherwise.

The co-infected branch (on the right-hand side) describes co-infection of the DIP-infected arrested cells (A). Cells become co-infected eclipse cells (CE) and co-infected infectious cells (CI) with an eclipse and infectious phase of length τ_E and τ_I, respectively. Co-infected infectious cells release a fraction $(1 - \varepsilon)$ of progeny DIPs and a fraction ε of progeny STV with production rate p. The DIPs also lose infectivity at rate c.

We use best fit parameter estimates from [12] where the infection kinetics of the 2009 pandemic influenza A/Québec/144147/09 (H1N1) virus strain, as published in [16], was re-analyzed (figure 2). Table S1 lists our base parameter values.

Table S1: **Base parameter values for infection with an influenza A (H1N1) pandemic strain [12].**

Parameter, *symbol*	Base value
Production rate, p	3.27×10^8 (PFU/mL) $\cdot$ h^{-1}
Fraction of progeny STV produced by co-infected cells, ε	0
Infection rate, β	2.44×10^{-7} (PFU/mL)$^{-1}$ $\cdot$ h^{-1}
Clearance rate, c	0.14 h^{-1}
Co-infection window length, τ_C	3 h
Eclipse length, τ_E	6.58 h
Infectious length, τ_I	46.8 h
Number of eclipse compartments, n_E	60
Number of infectious compartments, n_I	60
Rinsing factor, f_{resid}	8×10^{-5}
Adsorption time, t_{rinse}	1 h

When simulating an infection where the inoculum is incubated for one hour, then rinsed and replaced with fresh virus-free medium, when we assert that the sample contains 0.04 PFU/cell we mean that in simulating this infection with our kinetic mathematical model, 4% of cells will become infected by the inoculum under these conditions (1h incubation then rinse). As such, using 100x that same inoculum will correspond to an MOI of 4 PFU/cell. Just like in actual experiments, quantification of infectivity in our simulated inoculum is defined relative to both the specific cell type in which the infection proceeds (simulating a different cell type corresponds to changing cell-specific mathematical model parameters such as p, β, τ_E, τ_I, etc...), and also relative to the chosen infection methodology (rinsing after 1h incubation vs no rinse vs longer incubation period, etc...).

S5.3 Simulating infections

Infections are simulated by applying an initial inoculum (V_0) to a population of fully susceptible cells ($T_0 = 1$). The inoculum's STV concentration is characterized by the desired multiplicity of infection (MOI) and determined in the following way

$$V_0 = \frac{c}{\beta} \frac{\text{MOI}}{1 - \exp(-c t_{\text{rinse}})}. \tag{1}$$

If the initial inoculum contains DIPs, the DIP concentration (D_0) is determined in an analogous way. The initial inoculum is allowed to adsorb for a time t_{rinse}, before a rinse is simulated by reducing the particle concentrations by a factor f_{resid}.

S5.3.1 B&C reduction of STV yield assay

In the B&C assay [19], we are interested in the relative STV yield between an infection initiated with just the STV stock (stock-only infection), and infections initiated with both the STV stock plus dilutions of the DIP sample (stock-plus-sample infection). The stock-only infection is initiated with a STV MOI of 4 PFU/cell. The stock-plus-sample infections are initiated with the 4 PFU/cell STV stock, in addition to δ(8 DIP/cell) of the sample, where $0 \leq \delta \leq 1$ denotes the DIP dose. The B&C assay is simulated using base parameters unless otherwise noted. The 20 hpi STV yield from the stock-plus-sample infections are measured and normalized to that of the stock-only infection, in order to form the assay curve of relative STV yield as a function of DIP dose.

To estimate the percent error in the estimate of DIP MOI from B&C, the assay was performed for combinations of stock and sample at 10^{-3} PFU/cell–10^{1} PFU/cell and 10^{-3} DIP/cell–10^{1} DIP/cell, respectively. The sample DIP MOI was estimated from the reciprocal of the DIP dose which coincided with 37% relative STV yield, as in [20]. In some cases where the assay curve remained above, and did not cross the 37% relative STV yield, the DIP dose corresponding to 37% relative yield was linearly extrapolated. Explicitly, the percent errors were calculated with

$$\text{Percent error} = \left(\frac{\text{Estimated sample DIP MOI}}{\text{Actual sample DIP MOI}} - 1 \right)$$

S5.3.2 Nayak's time of (DIP) addition

To simulate Nayak's time of addition curve in [21], we are also interested in relative STV yield between a stock-only infection and stock-plus-sample infections, where the DIP-containing sample is applied at times subsequent to application of the STV stock. In the stock-plus-sample infections, the STV stock is adsorbed for 0.5 h, followed by rinsing. The time of STV stock application is defined as 0 h. After the STV stock application at 0 h, the sample (containing both DIPs and STV) is subsequently applied at various addition times, adsorbed for 0.5 h, rinsed, and the infection proceeds. One infection received the DIP sample 1 h before STV stock inoculation, corresponding to a −1 h time of addition. The STV yield is measured at 14 h after STV stock application, and normalized to the STV yield of the stock-only infection.

Using Engauge digitizer, we extracted data on the 14 h relative STV yield as a function of DIP addition time from [21]. Nayak et al. report the DIP MOI of their sample in units of PFU/cell, where the authors have assumed that DIPs are approximately 1,000 times less infectious than PFUs. As such, they estimated the sample DIP MOI as 4 DIP/cell (= 0.004 PFU/cell × 1000 DIP/PFU). Due to the uncertainty in both the concentrations of STV and DIPs, we allowed the LIB model to fit both the STV and DIP MOIs of the stock and sample, in addition to the model parameters. The best fit to these data yields a sum-of-squared residual (SSR) of 0.0032, where the set of best fit parameters is given in Table S2. Note that the sample's STV MOI was scaled by the stock STV MOI according to $\frac{0.68\ \text{PFU/cell}}{1\ \text{PFU/cell}} \times 0.004$ PFU/cell, and was not one of the fitted parameters. Importantly, we assumed that co-infected cells exclusively produce DIPs ($\varepsilon = 0$; base value), however our exploration of $\varepsilon \leq 10^{-3}$ allowed us to obtain fits with equivalent SSRs, with no impact on the extracted co-infection window or eclipse length.

The reliability of the co-infection window estimate was evaluated by assessing the sensitivity of the Nayak et al. time of addition curve to changes in infection kinetics. Figure S4 shows that the curve is only re-scaled in height, such that the location of the rise remains relatively unchanged when using a DIP-free inoculum of 1 PFU/cell: estimation of the co-infection window is mostly robust, within ±0.5 h. When the same sensitivity analysis is simulated using an inoculum with a higher STV MOI (4 PFU/cell), the estimate can be made even more robust, where the location of the rise is insensitive to most infection parameters, except for the eclipse length.

Table S2: **Best fit parameters to Nayak's time of DIP addition data.**

Parameter, *symbol*	Fitted value
Production rate, p	1.22×10^7 (PFU/mL) $\cdot$ h^{-1}
Fraction of progeny STV produced by co-infected cells, ε	0 (fixed)
Infection rate, β	9.83×10^{-2} (PFU/mL)$^{-1} \cdot$ h^{-1}
Clearance rate, c	0.04 h^{-1}
Co-infection window length, τ_C	3.5 h
Eclipse length, τ_E	7.11 h
Infectious length, τ_I	46.8 h (fixed)
Number of eclipse compartments, n_E	60 (fixed)
Number of infectious compartments, n_I	60 (fixed)
Rinsing factor, f_{resid}	8×10^{-5} (fixed)
Adsorption time, t_{rinse}	0.5 h (fixed)
Stock STV MOI	0.68 PFU/cell
Sample DIP MOI	2.30 DIP/cell
Sample STV MOI	0.0027 PFU/cell (computed)
Sum-of-squared residuals, SSR	0.0032

S5.3.3 STV peak drop between multiple-cycle and single-cycle infections of pandemic H1N1

We simulated the STV peak drop between a multiple-cycle (MC) and single-cycle (SC) infection that is caused by the presence of DIPs in the STV stock used to inoculate both infections, specifically in [16]. Experimentally, the MC infection is performed with a low STV MOI such that the chance of co-infection with DIPs is low. When DIP interference is negligible, we expect to observe a STV peak concentration that is characteristic of a DIP-free infection. The DIP-free STV peak in an MC infection is equivalent to that of an SC infection. Hence, instead of simulating the MC infection, we simply simulate a DIP-free SC infection, initiated with a 4 PFU/cell STV MOI and 1 h adsorption followed by rinsing, to obtain the DIP-free STV peak sampled at 30 hpi.

To obtain the STV peak drop, we simulate SC infections initiated with a 4 PFU/cell STV MOI and increasing DIP MOIs from 10^{-2} DIP/cell–4×10^1 DIP/cell. The 30 hpi STV peak from these DIP-containing SC infections are normalized to the DIP-free STV peak, which give us the STV peak drop. In these simulations, we used the base parameters in Table S1.

S5.3.4 Akkina's reversal of DIP-mediated interference

To simulate the reversal of DIP-mediated interference in [14], infections are initiated with initial inocula containing the STV and DIP MOIs given in figure S1. The 24 hpi STV yields were sampled.

We took published data from Table 3 of [14] which shows the STV yield, measured at 24 hpi, as a function of increasing STV MOI. A best fit to these data yielded an SSR of 0.528, with best fit parameters given in Table S3.

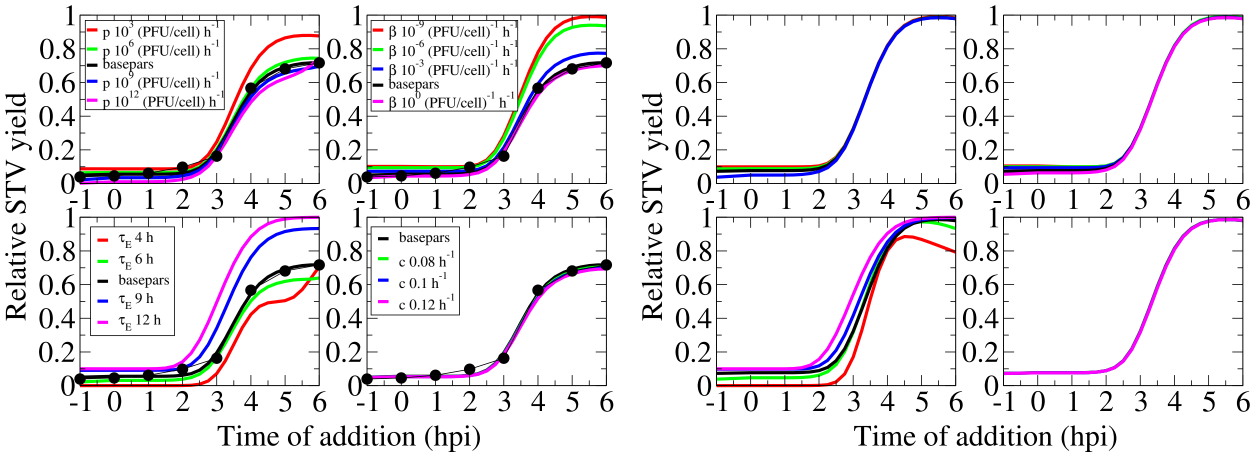

Figure S4: **Evaluating the reliability of the co-infection window estimated from the Nayak et al. assay.** Infections were simulated as in figure 7, with the best fit parameters as base values (thick black line), for simulated infections with a DIP-free STV inoculum of: (Left) 1 PFU/cell or (Right) 4 PFU/cell. The production rate, infection rate, clearance rate, and eclipse length are varied about their base value (clockwise from top left panel), for each of the two inocula. The original data from [21] are shown as black circles.

Table S3: **Best fit parameters to Akkina's reversal of DIP-mediated interference data.**

Parameter, *symbol*	Fitted value
Production rate, p	4.91×10^7 (PFU/mL) $\cdot$ h^{-1}
Fraction of progeny STV produced by co-infected cells, ε	0
Infection rate, β	2.43 (PFU/mL)$^{-1} \cdot$ h^{-1}
Clearance rate, c	0.1 h^{-1}
Co-infection window length, τ_C	4 h
Eclipse length, τ_E	6.58 h
Infectious length, τ_I	35 h
Number of eclipse compartments, n_E	30 (fixed)
Number of infectious compartments, n_I	30 (fixed)
Rinsing factor, f_{resid}	3.02×10^{-41}
Adsorption time, t_{rinse}	1 h (fixed)
STV and DIP MOI	as indicated (fixed)
Sum-of-squared residuals, SSR	0.528

References

[1] I.-C. Huang, W. Li, J. Sui, W. Marasco, H. Choe, and M. Farzan. Influenza A virus neuraminidase limits viral superinfection. J Virol. 2008;82(10):4834–4843. doi:10.1128/JVI.00079-08.

[2] K. Martin and A. Heleniust. Nuclear transport of influenza virus ribonucleoproteins: the viral matrix protein (M1) promotes export and inhibits import. Cell. 1991;67(1):117–130. doi:10.1016/0092-8674(91)90576-K.

[3] A. Kundu and D. P. Nayak. Analysis of the signals for polarized transport of influenza virus (A/WSN/33) neuraminidase and human transferrin receptor, type II transmembrane proteins. J Virol. 1994;68(3):1812–1818.

[4] P. U. Daniels and J. Edwardson. Influenza neuraminidase is delivered directly to the apical surface of MDCK cell monolayers. FEBS Lett. 1989;244(1):57–60. doi:10.1016/0014-5793(89)81161-5.

[5] A. C. Marriott and N. J. Dimmock. Defective interfering viruses and their potential as antiviral agents. Rev Med Virol. 2010;20(1):51–62. doi:10.1002/rmv.641.

[6] F. S. Heldt, T. Frensing, and U. Reichl. Modeling the intracellular dynamics of influenza virus replication to understand the control of viral RNA synthesis. J Virol. 2012;86(15):7806–7817. doi:10.1128/JVI.00080-12.

[7] F. T. Vreede, T. E. Jung, and G. G. Brownlee. Model suggesting that replication of influenza virus is regulated by stabilization of replicative intermediates. J Virol. 2004;78(17):9568–9572. doi:10.1128/JVI.78.17.9568-9572.2004.

[8] F. T. Vreede and G. G. Brownlee. Influenza virion-derived viral ribonucleoproteins synthesize both mRNA and cRNA in vitro. J Virol. 2007;81(5):2196–2204. doi:10.1128/JVI.02187-06.

[9] S. Heldt. Mathematical models of influenza A virus infection: From intracellular replication to virus growth in cell populations. Otto-von-Guericke Universität. Magdeburg, Germany; 2015. Available from: http://hdl.handle.net/11858/00-001M-0000-0026-A52B-8.

[10] T. Frensing, A. Pflugmacher, M. Bachmann, B. Peschel, and U. Reichl. Impact of defective interfering particles on virus replication and antiviral host response in cell culture-based influenza vaccine production. Appl Microbiol Biotechnol. 2014;98(21):8999–9008. doi:10.1007/s00253-014-5933-y.

[11] B. P. Holder, P. Simon, L. E. Liao, Y. Abed, X. Bouhy, C. A. A. Beauchemin, and G. Boivin. Assessing the in vitro fitness of an oseltamivir-resistant seasonal A/H1N1 influenza strain using a mathematical model. PLoS ONE. 2011;6(3):e14767. doi:10.1371/journal.pone.0014767.

[12] E. G. Paradis, L. Pinilla, B. P. Holder, Y. Abed, G. Boivin, and C. A. A. Beauchemin. Impact of the H275Y and I223V mutations in the neuraminidase of the 2009 pandemic influenza virus in vitro and evaluating experimental reproducibility. PLoS ONE. 2015;10(5):e0126115. doi:10.1371/journal.pone.0126115.

[13] P. F. Simon, M.-A. de La Vega, E. Paradis, E. Mendoza, K. M. Coombs, D. Kobasa, and C. A. A. Beauchemin. Avian influenza viruses that cause highly virulent infections in humans exhibit distinct replicative properties in contrast to human H1N1 viruses. Sci Rep. 2016;6:24154. doi:10.1038/srep24154.

[14] R. K. Akkina, T. M. Chambers, and D. P. Nayak. Mechanism of interference by defective-interfering particles of influenza virus: Differential reduction of intracellular synthesis of specific polymerase proteins. Virus Res. 1984;1(8):687–702. doi:10.1016/0168-1702(84)90059-5.

[15] T. Laske, F. S. Heldt, H. Hoffmann, T. Frensing, and U. Reichl. Modeling the intracellular replication of influenza A virus in the presence of defective interfering RNAs. Virus Res. 2016;213:90–99. doi:10.1016/j.virusres.2015.11.016.

[16] L. T. Pinilla, B. P. Holder, Y. Abed, G. Boivin, and C. A. A. Beauchemin. The H275Y neuraminidase mutation of the pandemic A/H1N1 virus lengthens the eclipse phase and reduces viral output of infected cells, potentially compromising fitness in ferrets. J Virol. 2012;86(19):10651–10660. doi:10.1128/JVI.07244-11.

[17] C. Seitz, T. Frensing, D. Höper, G. Kochs, and U. Reichl. High yields of influenza A virus in Madin-Darby canine kidney cells are promoted by an insufficient interferon-induced antiviral state. J Gen Virol. 2010;91(Pt 7):1754–1763. doi:10.1099/vir.0.020370-0.

[18] T. B. L. Kirkwood and C. R. M. Bangham. Cycles, chaos, and evolution in virus cultures: A model of defective interfering particles. Proc Natl Acad Sci USA. 1994;91(18):8685–8689.

[19] A. J. D. Bellett and P. D. Cooper. Some properties of the transmissible interfering component of vesicular stomatitis virus preparations. J Gen Microbiol. 1959;21(3):498–509. doi:10.1099/00221287-21-3-498.

[20] P. I. Marcus, J. M. Ngunjiri, and M. J. Sekellick. Dynamics of biologically active subpopulations of influenza virus: Plaque-forming, noninfectious cell-killing, and defective interfering particles. J Virol. 2009;83(16):8122–8130. doi:10.1128/JVI.02680-08.

[21] D. P. Nayak, K. Tobita, J. Janda, A. R. Davis, and B. K. De. Homologous interference mediated by defective interfering influenza virus derived from a temperature-sensitive mutant of influenza virus. J Virol. 1978;28(1):375–386.
